# Construction of Metabolic Molecular Classification and Immune Characteristics for the Prognosis Prediction of Ovarian Cancer

**DOI:** 10.1155/2022/2359349

**Published:** 2022-06-25

**Authors:** Kexin Wang, Hui He, Xue Feng

**Affiliations:** ^1^Department of Reproductive Medicine, The First Affiliated Hospital of Harbin Medical University, Harbin City, Heilongjiang Province, China 150001; ^2^Department of Laparoscopic Surgery, The First Affiliated Hospital of Dalian Medical University, Dalian City, Liaoning Province, China 116000

## Abstract

**Background:**

Ovarian cancer (OC) is a malignant tumor that seriously threatens women's health. Molecular classification based on metabolic genes can reflect the deeper characteristics of ovarian cancer and provide support for prognostic evaluation and the guidance of individualized treatment.

**Method:**

The metabolic subtypes were determined by consensus clustering and CDF. We used the ssGSEA method to calculate the IFN*γ* score of each patient. The CIBERSORT method was used to evaluate the score distribution and differential expression of 22 immune cells, and LDA was applied to establish a subtype classification feature index. The Kaplan-Meier and ROC curves were generated to validate the prognostic performance of metabolic subtypes in different cohorts. WGCNA was used to screen the coexpression modules associated with metabolic genes.

**Results:**

We obtained three metabolic subtypes (MC1, MC2, and MC3). MC2 had the best prognosis, and MC1 and MC3 had poor prognoses. Consistently, MC2 subtype had higher T cell lytic activity and lower angiogenesis, IFN*γ*, T cell dysfunction, and rejection scores. TIDE analysis showed that MC2 patients were more likely to benefit from immunotherapy; MC1 patients were more sensitive to immune checkpoint inhibitors and traditional chemotherapy drugs. The multiclass AUCs based on the RNASeq and GSE cohorts were 0.93 and 0.84, respectively. Finally, we screened 11 potential gene markers related to the metabolic characteristic index that could be used to indicate the prognosis of OC.

**Conclusion:**

Molecular subtypes related to metabolism are crucial to comprehensively understand the molecular pathological characteristics related to metabolism for OC development, explore reliable markers for prognosis, improve the OC staging system, and guide personalized treatment.

## 1. Introduction

Ovarian cancer (OC) is a malignant tumor that seriously threatens women's health. In 2020, 308,069 new cases of OC worldwide and 193811 deaths due to OC were estimated [1]. Because of the lack of effective screening methods and early diagnosis measures, 70% of OC patients are diagnosed in advanced stages (stage III or IV) [2], and 50% to 70% relapse within 2 years after treatment. At the same time, because OC is prone to recurrence, metastasis, and severe drug resistance, the 5-year overall survival rate of patients is only 25% to 35% [3, 4].

Increasingly understanding of the complexity of tumor biology improved the current knowledge of tumor metabolism. Changes in cell metabolism meet the needs of tissue internal environment homeostasis and growth. In cancer, malignant cells obtain metabolic adaptability through responding to various endogenous and exogenous signals. In the process of cancer progression, the metabolic characteristics and preferences of the tumor will change. Therefore, in the process of cancer development, how to use the metabolic changes in the tumor microenvironment (TME) to develop better treatment strategies has become the focus of our attention. The occurrence and development of OC is a complex multistage process in which the TME, particularly the tumor immune microenvironment (TIME), plays a vital role in the process of OC. The TME comprises cancer cells, surrounding blood vessels, extracellular matrix, signaling molecules, fibroblasts, and infiltrating immune cells, [5]. Using the negative regulatory mechanism of the body's immune system, tumor cells can regulate the TME. A full range of immunosuppressive states can be used to counter the body's antitumor immunity [6, 7]; individual differences in the efficacy of tumor immunotherapy are closely related to immunosuppression in the TME [5]. Stromal cells and immune cells infiltrating tumor tissues constitute the main components of the dynamic network of the TME. Research has confirmed that the microenvironment of OC is closely related to the growth and proliferation of tumor cells, formation of new blood vessels, tumor invasion and metastasis, immunosuppression, and drug resistance [8, 9].

Currently, clinical pathological staging is commonly used to assess the prognosis of patients with OC. Patients with early (stage I-II) epithelial OC shows a significantly better prognosis than that those at advanced stage (stage III-IV), with an increased 5-year survival rate up to 60%~90% after operation, according to the International Federation of Obstetrics and Gynecology (FIGO) staging standards [10]. However, an increasing number of studies have shown that OC is a group of highly heterogeneous diseases with different molecular phenotypes, pathogeneses, and prognoses. A single FIGO staging or WHO histological classification has an effect on the prognosis. The predictive value is very limited [11]. In 2004, based on the pathomorphology and molecular genetic analysis of OC, Shih and Kurman [12] established a dualistic model and classified OC into type I and type II. Although traditional WHO histological classification and binary classification have far-reaching significance in the research process of OC, with the gradual in-depth research of OC at the molecular genetic level in recent years, its limitations have become increasingly obvious. According to the WHO classification, the reproducibility between observers is poor, particularly for the prognostic evaluation of advanced OC, and it cannot be used as an independent factor to predict the prognosis of OC [13].

With the development of gene chip and high-throughput sequencing technologies, based on the big data of the GEO and TCGA databases, the comprehensive and systematic analyses of tumor-related genes and their regulatory mechanisms using bioinformatics methods are an important part of the current tumor genomics group [14]. Metabolic disorders, as an essential characteristic of tumors [15], have an overall impact on various tumor biological behaviors, including occurrence, development, metastasis, and recurrence [16, 17].

In the present study, we have established a molecular classification model of OC based on metabolic characteristics and have constructed the immune characteristic index of each subtype to supplement the deficiencies of the clinical staging system. Our study findings will provide research ideas and a theoretical basis for prognostic estimation and individualized treatment of OC patients.

## 2. Materials and Methods

### 2.1. Expression Profile Data Preprocessing and Metabolism-Related Genes

TCGA-Ovarian cancer cohort containing RNASeq data and clinical information was downloaded from The Cancer Genome Atlas database (TCGA, https://portal.gdc.cancer.gov/). ICGC cohort with OC samples containing RNASeq data and clinical information was obtained from International Cancer Genome Consortium database (ICGA, https://dcc.icgc.org/). Microarray chip data of GSE26193, GSE30161, GSE63885, and GSE9891 (including survival time) were obtained from Gene Expression Omnibus database (GEO, https://www.ncbi.nlm.nih.gov/geo/). Fragments per kilobase million (FPKM) of RNASeq data was converted to transcript per million (TPM) format. OC samples without survival status and survival time were eliminated. For GSE data, probes were converted to gene symbol and the probes that correspond to multiple genes were removed. The median value was selected when one gene had multiple probes.

“Remove Batch Effect” function in the limma package [18] was used to remove batch effects among different cohorts (Supplementary Figure S1). The expression profiles of GSE26193, GSE30161, GSE63885, and GSE9891 were combined (hereinafter referred to as GSE cohort). The RNASeq data of TCGA and ICGC cohorts were combined (hereinafter referred to as RNASeq cohort). After preprocessing, 465 and 511 OC samples were remained in RNASeq and GSE cohorts, respectively. In the metabolism-related gene sets from previous studies [19], a total of 2752 genes were selected.

### 2.2. Classification of OC Subtypes

First, single-factor analysis was used to screen prognostic-related “metabolic genes.” OC samples were clustered using the ConsensusClusterPlus R package [20], and stable clustering results were determined according to the cumulative distribution function (CDF) and CDF delta area curve, and the metabolism of OC was constructed using the selected metabolic genes. ConsensusClusterPlus is a method based on resampling to verify the rationality of clustering. The resampling method can disrupt the original cohort. Thus, cluster analysis is performed on each resampled sample and then comprehensively evaluated. The results of subcluster analysis provide an assessment of consistency (Consensus). The main purpose was to evaluate the stability of clustering.

### 2.3. Single-Sample Gene Set Enrichment Analysis (ssGSEA)

ssGSEA is an extension of gene set enrichment analysis (GSEA) [21]. Each ssGSEA enrichment score represents the absolute degree of enrichment of genes in a specific gene set in the sample. The gene expression values of a given sample were sorted and normalized, and the empirical cumulative distribution function (ECDF) of the genes in the signature and the remaining genes was used to generate an enrichment score. To analyze the Th1/IFN*γ* expression differences in metabolic subtypes, we used the ssGSEA method to calculate the IFN*γ* score of each patient.

### 2.4. Features of Immune Infiltration

To study the immune characteristics between different metabolic subtypes, we used the CIBERSORT method to evaluate the score distribution and differential expression of 22 immune cells in the RNASeq cohort. CIBERSORT [22] is a tool for deconvolution of the expression matrix of immune cell subtypes based on the principle of linear support vector regression. Using the CIBERSORT function, the tissue transcriptome sequencing expression profile was statistically analyzed, and the deconvolution method was used to denoise and remove the unknown mixture content to estimate the relative proportion of 22 immune cell subpopulations. According to the expression profile data of each sequenced sample, the relative expression of specific genes was analyzed to predict the proportion of 22 types of immune cells.

### 2.5. Prediction of Immunotherapy/Chemotherapy and Construction of the Subtype Characteristic Index

To compare the similarities between different metabolic subtypes and the GSE91061 cohort (melanoma cohort receiving anti-PD-1 and anti-CTLA-4 treatment) between immunotherapy patients, we adopted a subclass mapping method (SubMap analysis) [23]. This methodology allows to compare the similarity of expression profiles between two groups. The Bonferroni-corrected *P* values were employed to determine the similarity between two groups, where the more obvious significance represented the higher similarity of two them. R package of pRRophetic [24] was applied to calculate the biochemical half maximal inhibitory concentration (IC50) of traditional chemotherapy drugs (cisplatin, vinorelbine, embelin, and pyrimethamine) in different subtypes.

To better quantify the immune characteristics of patients in different sample cohorts, we used linear discriminant analysis (LDA) to establish a subtype classification feature index.

### 2.6. Weighted Correlation Network Analysis (WGCNA)

We selected the RNASeq cohort (MAD > 50%), used the R software package WGCNA [25] to cluster the samples, and screened the coexpression modules of metabolic genes. The coexpression network conforms to the scale-free network (correlation coefficient > 0.85). Based on TOM, we used the average-linkage hierarchical clustering method to cluster genes (gene network module^min^ = 80). Using the dynamic shear method, we calculated the eigengenes and merged the modules into a new module (height = 0.25; DeepSplit = 3; minModuleSize = 80).

### 2.7. Statistical Analysis

Statistical analysis was performed using R software (v4.1). To compare the measurement data between groups, one-way analysis of variance or *t* test was used for data that conform to a normal distribution, the Kruskal-Wallis *H* test or Mann-Whitney *U* test was used for nonnormal data; chi-squared test or Fisher's exact probability method was used for count data. In difference analysis, test level *α* = 0.05 was determined and data with *P* < 0.05 was selected for further analysis. Log-rank test was conducted in Kaplan-Meier survival analysis and Cox regression analysis. ANOVA test was performed among three groups. In all statistical analysis, *P* < 0.05 was considered as significant.

## 3. Results

### 3.1. Molecular Subtype Based on Metabolic Gene Construction

**Figure 1:**
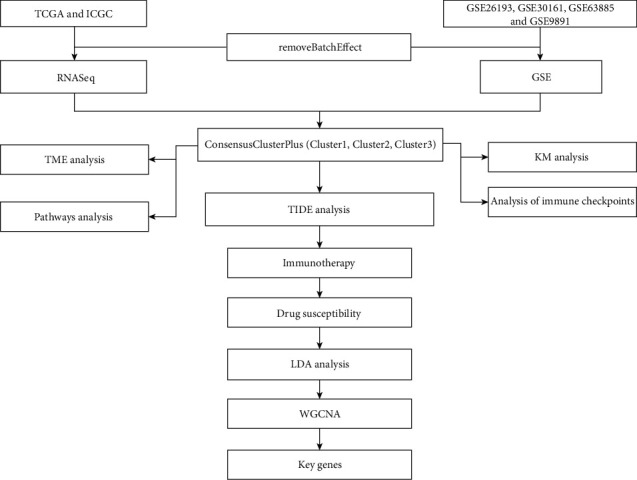
The flow chart of this study.

**Figure 2:**
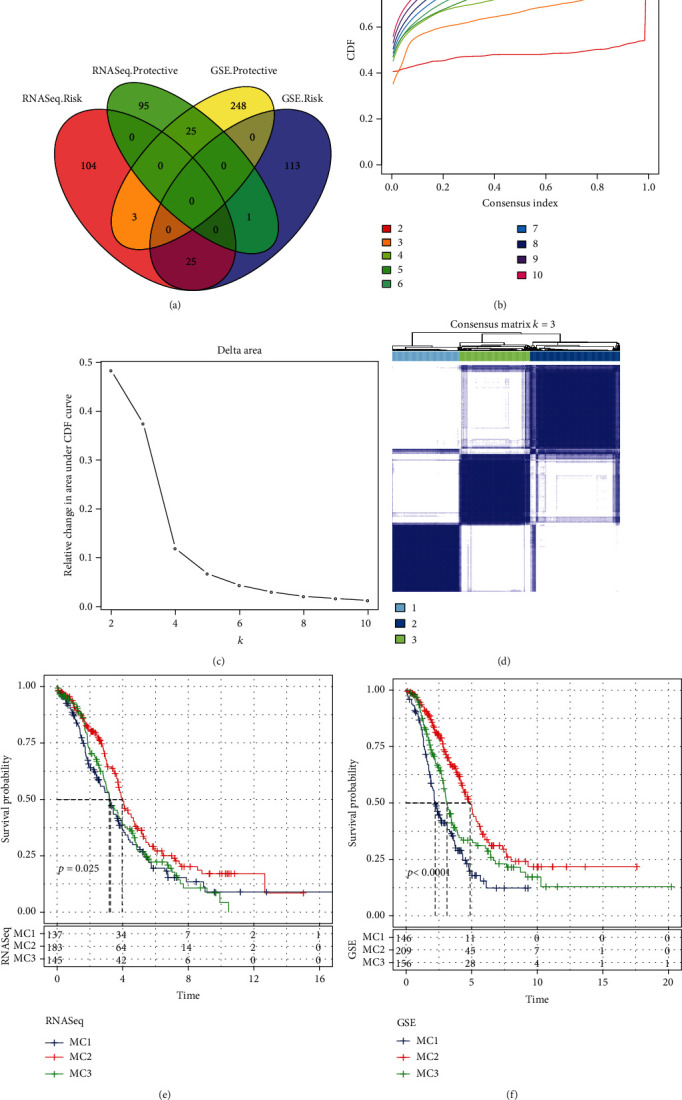
Metabolism cluster in OV. (a) Intersection Venn diagram of metabolic genes with significant prognosis in the two cohorts. (b and c) RNASeq cohort sample CDF curve and CDF delta area curve and delta area curve of consensus clustering, indicating the relative change in the area under the cumulative distribution function (CDF) curve for each category number *k* compared with *k* – 1. The horizontal axis represents category number k, and the vertical axis represents the relative change in the area under the CDF curve. (d) Consensus *k* = 3. Sample clustering heat map. (e) KM curve of the prognosis of the three subtypes in the RNASeq cohort. (f) KM curve of the prognosis of the three subtypes in the GSE cohort.

The work flow of this study is shown in Figure 1. We first calculated the univariate analysis of metabolic genes from the two cohorts. Univariate survival analysis showed that 253 genes (RNASeq data) and 415 genes (GSE microarray data) were associated with prognosis, respectively. The number of overlapped genes was 50 (Figure 2(a) and Supplementary Table S1), indicating that the consistency of metabolic genes is poor among cohorts of different platforms. Therefore, we used the 50 metabolic genes that were identified as prognostic-related for subsequent analysis (log-rank test, *P* < 0.05).

In the RNASeq cohort, 465 OV samples were clustered by consensus clustering (ConsensusClusterPlus), and the optimal number of clusters was determined by the cumulative distribution function (CDF). Using the CDF delta area curve, we observed relatively stable clustering when the cluster was selected as 3 (Figures 2(b) and 2(c)). Ideally, an optimal cluster could be determined in the situation when a CDF curve mildly descending and the area under CDF curve maintaining a high value. Simultaneously, a small number of clusters were prior to be chosen for effectively subtyping samples. Therefore, we chose *k* = 3 to obtain three metabolic subtypes (metabolism cluster, MC) with different expression patterns of the 50 genes (Figure 2(d) and Supplementary Figure S2). Further analysis of the prognostic characteristics of these three metabolic subtypes showed that the prognosis of MC1 and MC3 was poor, and the prognosis of MC2 was good, with significant differences (log-rank test, *P* = 0.025, Figure 2(e)). Additionally, we observed the same phenomenon in the GSE queue using the same method (log-rank test, *P* < 0.0001, Supplementary Figure S2, S4 and Figure 2(f)). These results indicate that the three molecular subtypes based on metabolic genes are replicable in different research cohorts.

### 3.2. Expression of Chemokines and Immune Checkpoint Genes in Metabolic Subtype

**Figure 3:**
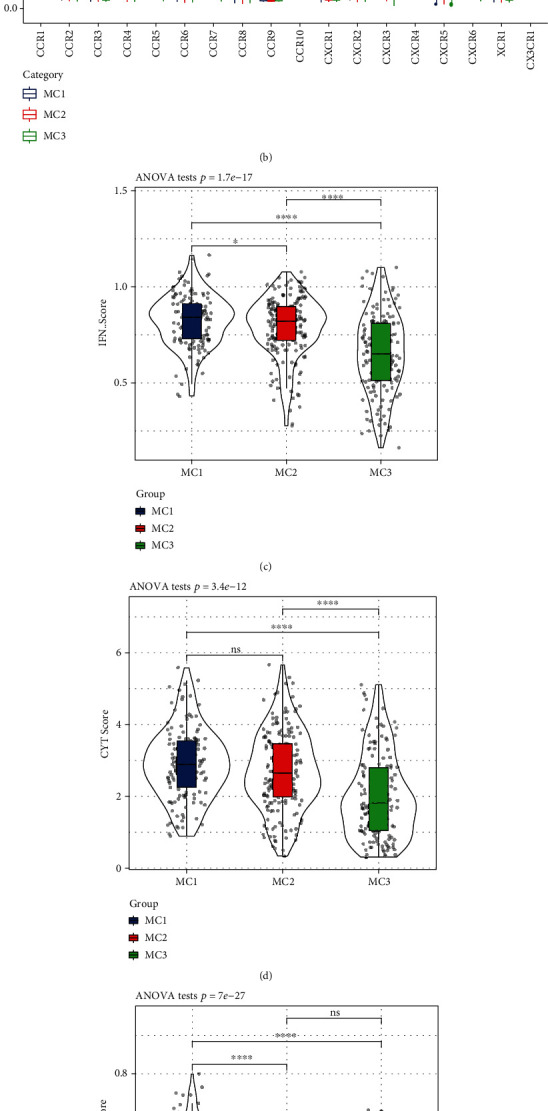
(a) Difference in the expression and distribution of chemokines in the RNASeq cohort. (b) Difference in the expression and distribution of chemokine receptors in the RNASeq cohort. (c) Difference in the distribution of IFN*γ* scores among the different subgroups in the RNASeq cohort. (d) Differences in the immune T cell lysis activity among the different subgroups. (e) Differences in the angiogenesis scores among the different subgroups. (f) Differences in the expression and distribution of immune checkpoint genes in the RNASeq cohort; the significance is statistically tested using analysis of variance. ^∗^*P* < 0.05, ^∗∗^*P* < 0.01, ^∗∗∗^*P* < 0.001, and ^∗∗∗∗^*P* < 0.0001.

To analyze the differences in the expression of chemokines in the three metabolic subtypes, we calculated the differences in these genes in the RNASeq cohort (Figure 3(a)). Twenty-seven of the 33 chemokines (81.82%) vary in the subtypes. Significant differences were found in the immune system, suggesting that the degree of immune cell infiltration of different metabolic subtypes may be different. These differences may lead to differences in tumor progression and immunotherapy effects. Additionally, we calculated and compared the expression of chemokine receptor genes in the metabolic subtypes (Figure 3(b)) and found that 16 (88.89%) of the 18 chemokine receptor genes were expressed in the metabolic subtypes, with significant differences (*P* < 0.05).

To analyze the differences in Th1/IFN*γ* expression among the three metabolic subtypes, we extracted Th1/IFN*γ* gene signatures from previous studies [26], calculated the IFN*γ* score of each patient using the ssGSEA method, and observed each subtype. Significant differences were found in the IFN*γ* scores among the three groups. The MC1 subgroup had higher IFN*γ* scores, while the MC2 and MC3 subgroups had the lower IFN*γ* scores (Figure 3(c)).

Furthermore, according to a previous study by Rooney et al. [27], the average value of the GZMA and PRF1 expression levels was used to evaluate the intratumoral immune T cell lysis activity of each patient. Significant differences were found among the three subgroups (Figure 3(d)). Interestingly, MC1 and MC2 had the highest immune T cell lytic activity, while MC3 had the lowest immune T cell lytic activity.

The angiogenesis-related gene set obtained from a previous study [28] was used to evaluate the angiogenesis score of each patient. Significant differences were found among the subgroups (Figure 3(e)). The angiogenesis score of MC1 was significantly higher than that of MC2 and MC3.

Furthermore, we obtained 47 immune checkpoint-related genes from previous studies [26] and analyzed their differences among the metabolic subtypes. Forty-two (89.36%) genes showed significant differences in the metabolic subtypes (Figure 3(f)), and most of the immune checkpoint-related genes were expressed at significantly higher levels in MC1 and MC2 than in MC3. Among them, T cell exhaustion markers, such as LAG3, CTLA4, PDCD1, CD276, and HAVCR2, were highly expressed in MC1 subtype. Thus, different subgroups may have different responses to immunotherapy.

### 3.3. Immune Characteristics and Pathway Characteristics among the Metabolic Subtypes

**Figure 4:**
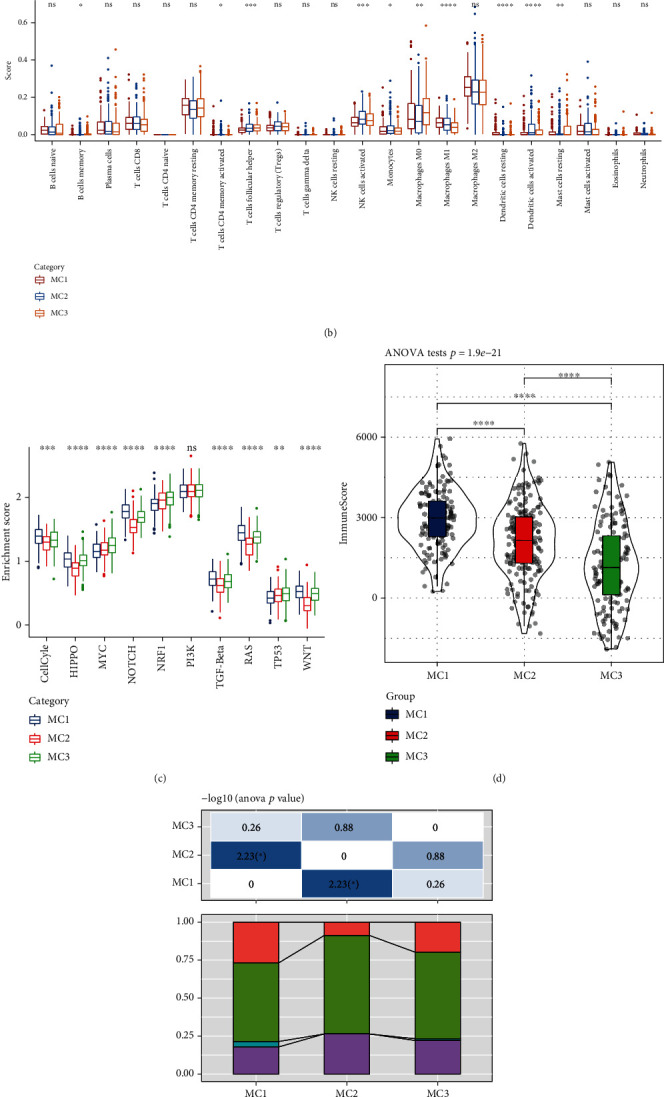
(a) Ratio of 22 immune cell components in the different subgroups. (b) Differences in 22 immune cell components of samples among the different subgroups. (c) Differences in the scores of 10 pathways related to tumor abnormalities among the different subgroups. (d) Differences in the immune infiltration scores among the different subgroups. (e) Comparison of the three metabolic molecular subtypes with the previous six pancancer metabolic molecular subtypes. ANOVA test was conducted among three groups. ns: no significance. ^∗^*P* < 0.05, ^∗∗^*P* < 0.01, ^∗∗∗^*P* < 0.001, and ^∗∗∗∗^*P* < 0.0001.

In the RNASeq cohort, the CIBERSORT method was used to evaluate the scores of 22 immune cells in each sample, observe the distribution of these immune cell scores in the three subgroups and the difference results (Figures 4(a) and 4(b)), and observe the immune cell scores in the three subgroups. Overall, significant differences were found in the immune characteristics among the subgroups. Activated NK cells and M1 macrophages were significantly highly expressed in the MC2 subtypes, immune infiltration analysis showed that MC1 had the highest immune microenvironment infiltration, and MC3 had the lowest immune infiltration score (Figure 4(d)).

We analyzed the differences in the 10 oncogenic pathways of the three subgroups in the previous study [29], revealing that 9 of the 10 pathways showed significant differences among the subtypes, including cell cycle, HIPPO, NOTCH, TGF-Beta, RAS, WNT, and other pathways, with low scores in the MC2 subtype (Figure 4(c)).

To observe the relationship among the three metabolic molecular subtypes and six previous pancancer immunophenotypes, we extracted and compared the molecular subtype data of these samples from previous studies [30]. Significant differences were found in immunophenotyping (Figure 4(e)), but no difference was observed between the survival curves of OV samples in pancancer immunophenotyping. This result suggested that the three subtypes can be used as a supplement to the six subtypes in the previous study.

### 3.4. MC1 Subtype May Have T Cell Depletion in the Immune Microenvironment

**Figure 5:**
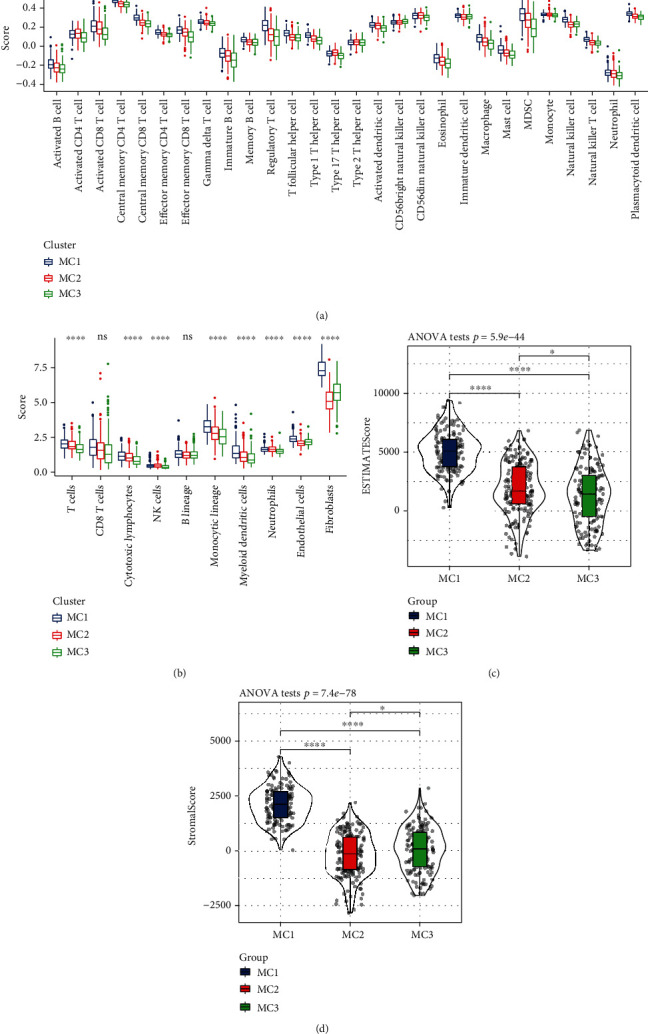
(a) Comparison of the subtypes of 28 immune cell scores evaluated by ssGSEA. (b) Comparison of the subtypes of 10 immune cell scores evaluated by MCP-Counter. (c) Comparison of the subtypes evaluated by the ESTIMATE of StromalScore score. (d) Comparison of the subtypes by the ESTIMATE score evaluated by ssGSEA Subtype comparison. ANOVA test was conducted among three groups. ns: no significance. ^∗^*P* < 0.05, ^∗∗^*P* < 0.01, ^∗∗∗^*P* < 0.001, and ^∗∗∗∗^*P* < 0.0001.

Based on the RNASeq data, we used MCP-Counter to analyze the scores of 10 immune cells, the ssGSEA function of GSEA to analyze the scores of 28 immune cells [31], and ESTIMATE to evaluate the overall immune microenvironment infiltration score. MCP-Counter analyzed 10 immune cell scores, and 8 were higher in MC1 subtype (Figure 5(a)). ssGSEA analyzed 28 immune cell scores, and MC1 had higher immune scores (Figure 5(b)). ESTIMATE evaluation revealed that the scores of the three metabolic subtypes were consistent with MCP-Counter and ssGSEA (Figures 4(d) and 5(c) and 5(d)). This finding combined with the previous immune checkpoint analysis further confirmed that the MC1 subtype may show T cell exhaustion.

### 3.5. MC2 Metabolic Subtype May Benefit from Immunotherapy

**Figure 6:**
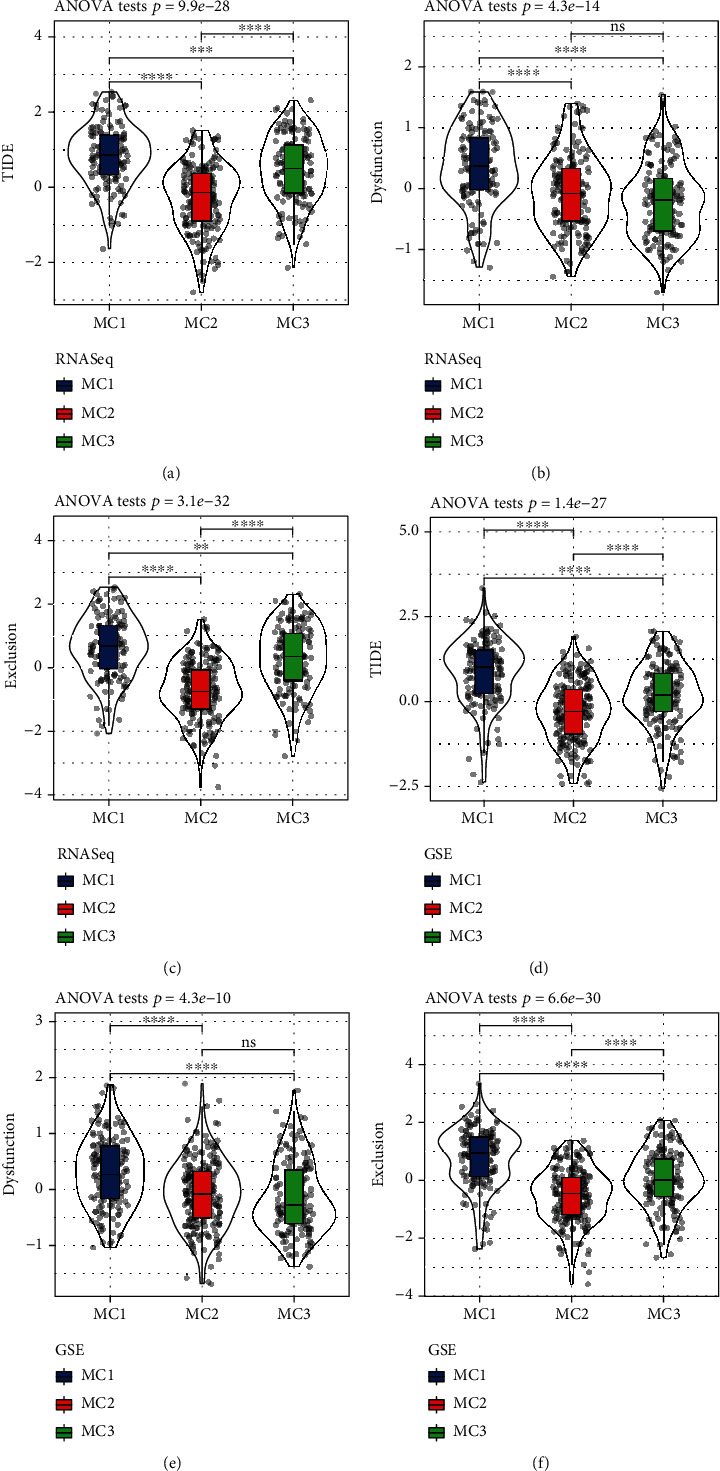
(a) TIDE score difference among the metabolic subtypes of RNASeq. (b) T cell dysfunction score difference among the metabolic subtypes of RNASeq. (c) T cell rejection score difference among the metabolic subtypes of RNASeq. (d) GSE TIDE score difference among the metabolic subtypes. (e) T cell dysfunction score difference among the HCCDB18 metabolic subtypes. (f) T cell rejection score difference among the GSE metabolic subtypes. (g) TIDE score difference among the GSE metabolic subtypes. ANOVA test was conducted among three groups. ns: no significance. ^∗∗^*P* < 0.01, ^∗∗∗^*P* < 0.001, and ^∗∗∗∗^*P* < 0.0001.

To evaluate the potential clinical effects of immunotherapy among the different metabolic subtypes, we used TIDE. The higher is the TIDE prediction score, the higher is the possibility of immune escape, indicating that the patient is less likely to benefit from immunotherapy. In the RNASeq cohort, the TIDE scores of MC1 and MC3 (*P* < 0.01) were significantly higher than those of MC2, suggesting that MC2 can benefit from immunotherapy more than MC1 and MC3 (Figure 6(a)). Comparing the predicted T cell dysfunction scores and T cell rejection scores among the different metabolic molecular subtypes (Figures 6(b) and 6(c)) revealed that the T cell dysfunction scores predicted by MC2 and MC3 were lower than those predicted by MC1. In the comparison of the predicted T cell rejection scores, MC2 had the lowest T cell rejection score, while MC1 had the highest T cell rejection score. We also observed similar results on the GSE cohort (Figures 6(d)–6(f)).

**Figure 7:**
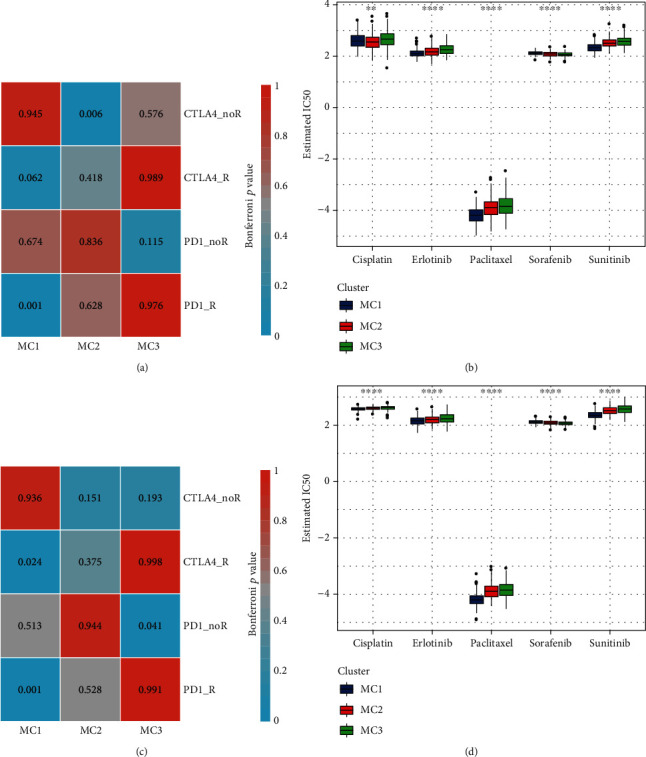
(a) SubMap analysis in RNASeq cohort shows that MC1 is more sensitive to CTLA4 (Bonferroni-corrected *P* < 0.05). (b) Box plots of the estimated IC50 in RNASeq cohort. (c) SubMap analysis in GSE cohort shows that MC1 is more sensitive to CTLA4 (Bonferroni-corrected *P* < 0.05). (d) Box plots of the estimated IC50 in GSE cohort. noR and R indicate no response and responsive to immunotherapy, respectively. ANOVA test was conducted in (b) and (d). ^∗∗^*P* < 0.01 and ^∗∗∗∗^*P* < 0.0001.

We further used the subclass mapping method to compare the similarities among the three metabolic subtypes and immunotherapy patients in the GSE91061 cohort. The cohort analysis revealed that the MC1 subtype was more sensitive to CTLA4 and PD1 inhibitors than the other two subtypes (Figures 7(a) and 7(c)). We also analyzed the response of the different subtypes to the traditional chemotherapy drugs cisplatin, paclitaxel, embelin, and sorafenib and found that the MC1 subtype was more sensitive to these four drugs than the other subtypes (Figures 7(b) and 7(d)).

### 3.6. LDA and Construction of the Metabolic Subtype Characteristic Index

**Figure 8:**
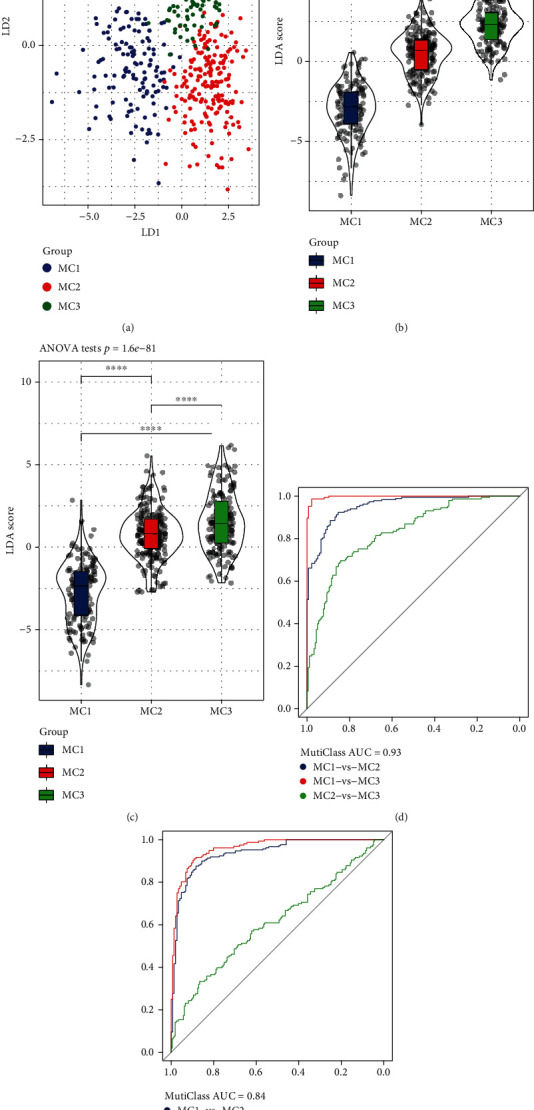
(a) Relationship between the first two features in the RNASeq metabolic feature index and metabolic subtypes. (b) Differences in the immune feature index among different subtypes in the RNASeq cohort. (c) Differences in the immune feature index among different subtypes in the GSE cohort. (d) ROC curve of the immune characteristic index in the RNASeq cohort. (e) ROC curve of the immune characteristic index in the GSE cohort. ANOVA test was conducted among three groups. ^∗∗∗∗^*P* < 0.0001.

Considering that different subtypes have different molecular characteristics, we better quantified the immune characteristics of patients in different sample cohorts using linear discriminant analysis (LDA) to establish a subtype classification feature index. LDA can be used as a supervised dimensionality reduction technology that is often suitable for multiple conditions; specifically, we used the 50 prognostic-related features in the RNASeq cohort, performed z-transformation on each feature, and used Fisher's LDA optimization standard to specify the centroid of each group. We dispersed as much as possible and found a linear combination A that maximizes the between-class variance of A relative to the within-class variance. The first two features of the model clearly distinguished among samples of the different subtypes (Figure 8(a)). Based on the LDA model, we calculated the subtype feature index of each patient in the RNASeq cohort. Significant differences were found in the feature index of the different subtypes (Figure 8(b)). ROC analysis showed the classification performance of the feature index in the different subtypes (Figure 8(d)). The category comprehensive forecast AUC was 0.93. Applying the metabolic subtype feature index to the GSE cohort, we observed that the results were similar to those of the RNASeq cohort. Significant differences were found in the feature index of the different subtypes (Figure 8(c)). ROC analysis showed that the comprehensive AUC was 0.84 (Figure 8(e)).

### 3.7. Identification of “Brown Module” of the Metabolic Characteristic Index and Prognostic Genes

**Figure 9:**
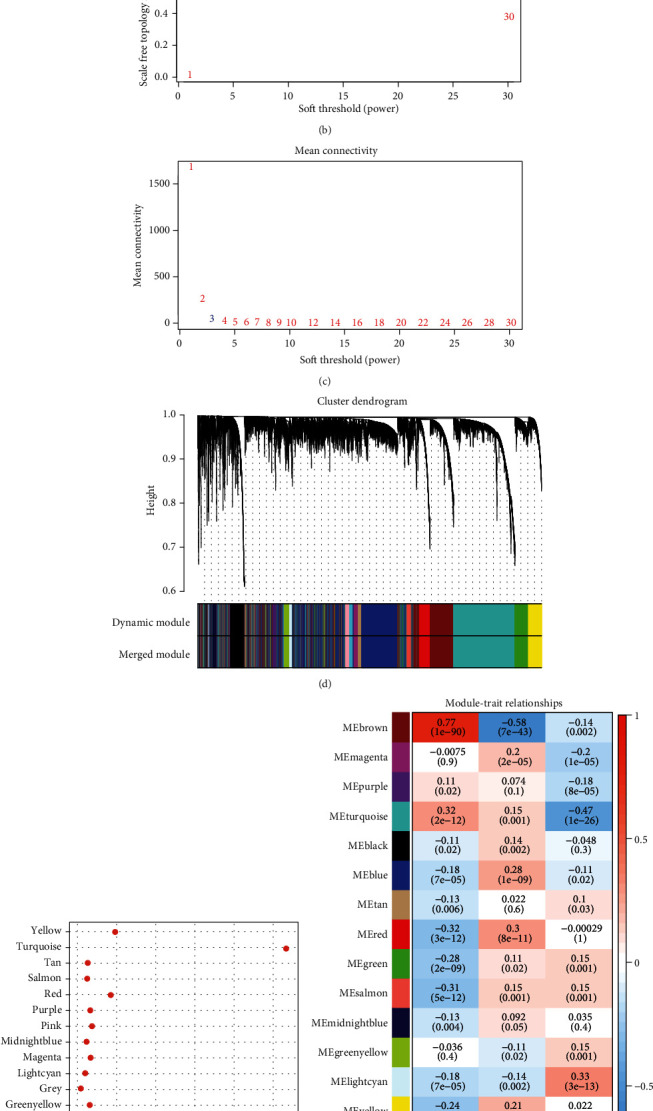
(a) Clustering tree of each sample. (b) Analysis of the scale-free fit index for various soft-thresholding powers (*β*). (c) Analysis of the mean connectivity for various soft-thresholding powers. (d) Dendrogram of all differentially expressed genes/lncRNAs clustered based on a dissimilarity measure (1-TOM). (e) Statistical analysis of the number of genes in each module. (f) Correlation between each module and subtype.

We used the R software package WGCNA to identify the coexpression modules of these immune genes. Specifically, we chose the RNASeq expression profile cohort, first clustered the samples (Figure 9(a)), chose a soft threshold of 3, and screened the coexpression modules. The coexpression network conforms to the scale-free network; that is, the logarithm log(k) of the node with connection degree *k* and the logarithm log(*P*(*k*)) of the probability of the node appearing are negatively correlated, and the correlation coefficient is greater than 0.85. To ensure that the network was a scale-free network, we chose *β* = 3 (Figures 9(b) and 9(c)). In the next step, the expression matrix was converted into an adjacency matrix, and then, the adjacency matrix was converted into a topological matrix. Based on TOM, we used the average-linkage hierarchical clustering method to cluster genes according to the standard of the hybrid dynamic shearing tree and set the minimum number of genes in a gene network module as 150. After determining the gene modules using the dynamic shear method, we calculated the eigengenes of each module in turn, performed cluster analysis on the modules, and merged the modules that were closer to each other into a new module in the conditions of height = 0.25, DeepSplit = 2, and minModuleSize = 150. Seventeen modules were obtained (Figure 9(d)). Notably, the gray module was a gene set that could not be aggregated into other modules. The transcripts of each module were counted (Figure 9(e)). We then analyzed the correlation between each module and MC1, MC2, and MC3 to figure out the key modules. The result displayed that brown module was highly correlated with MC1 and MC2 with correlation coefficients of 0.77 and -0.58 (*P* = 1e − 90 and *P* = 7e − 43), respectively (Figure 9(f)). In addition, turquoise module was closely associated with MC3 (*R* = −0.47, *P* = 1e − 26).

**Figure 10:**
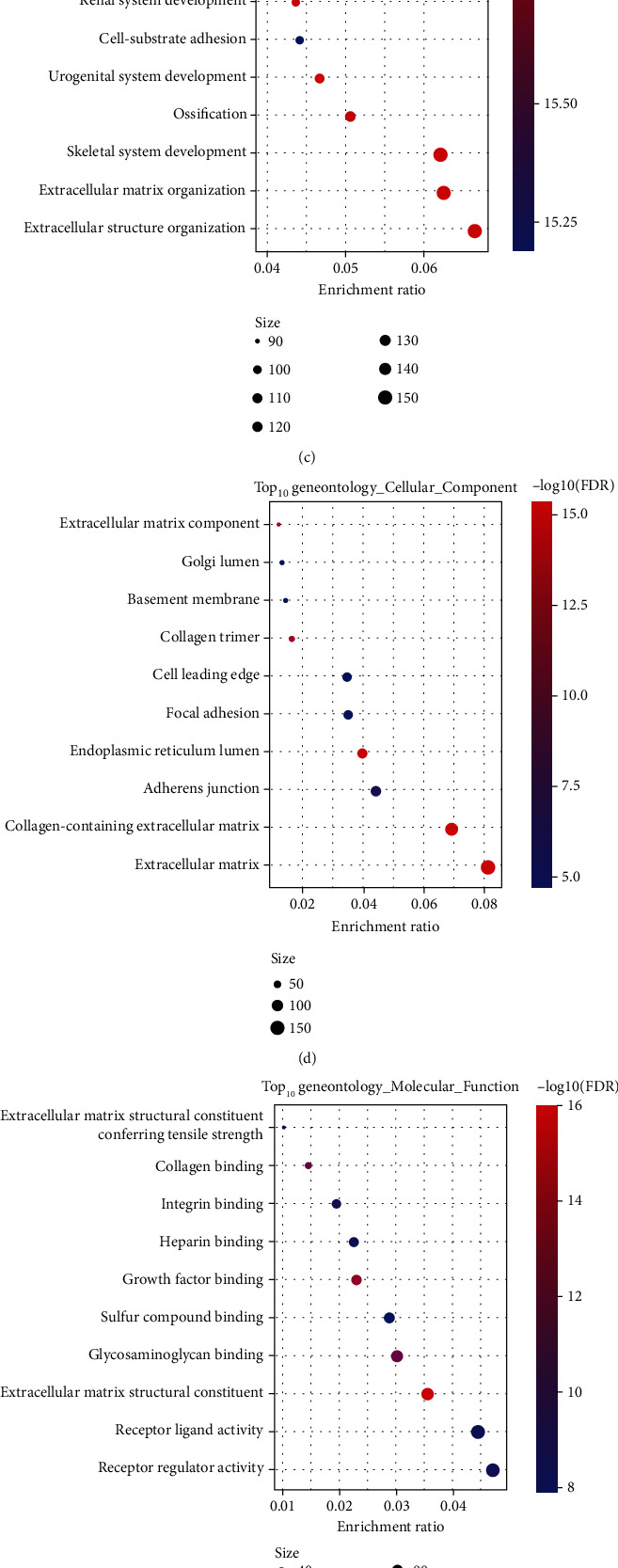
(a) Correlation analysis of the LDA score and metabolic characteristic index. (b) Prognostic correlation of modules related to the immune characteristic index. Log-rank test was conducted. (c) Functional enrichment analysis of brown module genes.

We calculated the correlation between the feature vector of these 17 modules and the metabolic feature index (Figure 10(a)), from which 17 blocks showed a significant correlation with the immune feature index. Furthermore, we selected modules that were significantly related to the metabolic characteristic index for prognostic analysis (Figure 10(b)). We further screened the brown module based on our defined metabolic molecular subtypes and the relationship between the module and prognosis. Based on the module feature vector correlation coefficient > 0.8 and significant prognostic genes as the module's hub genes, as well as selecting *P* < 0.05 as a threshold to filter, we finally identified 11 key genes in the brown module. These 11 genes were *GALNT5*, *COL8A1*, *FZD1*, *CCN4*, *ZEB1*, *ZCCHC24*, *CLMP*, *LUM*, *FBN1*, *ITGA11*, and *ZNF469*. To understand whether the expression of 11 genes was associated with the immune infiltration, we performed the Pearson correlation analysis between their expression and the immune score in OC as well as other 32 cancer types (Supplementary Figure S5). In ovarian cancer, the expression of all 11 genes was significantly associated with immune score. The correlation was also found in other cancer types such as pancreatic adenocarcinoma (PAAD), colon adenocarcinoma (COAD), rectum adenocarcinoma (READ), prostate adenocarcinoma (PRAD), and bladder urothelial carcinoma (BLCA).

We also divided patients into high and low expression groups based on gene expression and analyzed differences in the prognosis between the high and low gene expression groups (Supplementary Figure S6). Furthermore, the survival curves of the *GALNT5*, *FZD1*, *ZEB1*, *ZCCHC24*, *FBN1*, and *ITGA11* genes were significantly different (*P* < 0.05). Next, we used the clusterProfiler package to enrich the genes of the brown module (Figures 10(c)–10(f)) and observed the interaction of our brown module with ECM receptors, cell adhesion molecules (CAMs), proteoglycans in cancer, and the PI3K-Akt signaling pathway. Thus, the tumor process is closely related.

## 4. Discussion

In recent years, researchers have focused on elucidating the pathogenesis and epidemiology of OC. Targeted diagnosis and treatment that fully consider the molecular heterogeneity of malignant tumors have emerged as the development direction of malignant tumors in the future, and precise classification based on the molecular level is the basis for individualized diagnosis and treatment. Tumors are systemic diseases with multifactor origins and multistep development. Tumors are highly heterogeneous at the molecular level, with tumors of the same histological morphology showing inconsistent molecular genetic changes. Additionally, the growth of malignant tumors must cooperate with tumor-related stromal cells and the microenvironment required by tumors. Therefore, studies should establish a prognostic evaluation model for OC with clinicopathological characteristics and gene clusters. The model not only essentially analyzes and types the occurrence of OC but also effectively improves the prediction accuracy of the prognostic evaluation model.

In a previous study, 285 cases of ovarian endometrioid carcinoma and serous carcinoma have been profiled for miRNA gene expression, and molecular subtype has been classified by the K-means method and 6 subtypes (C1~C6) were determined. Subtypes are predictive of high-grade serous OC prognosis [32]. In 2011, the TCGA team analyzed the whole genome of a large sample of OC and found that 96% of patients with high-grade serous OC had mutations in TP53, while few mutations of NF1, BRCA1, BRCA2, RB1, and CDK12 were observed, but they were significant. Serous OC is classified into immunoreactive, proliferative, differentiated, and mesenchymal cell types according to mRNA expression, and this type is related to the prognosis; the immunoreactive type has the best prognosis, and the leaf cell type has the worst prognosis [33]. Afterward, Jönsson et al. further combined clinicopathological factors with gene expression characteristics based on TCGA classification, used de novo sequencing to classify high-grade serous OC into 4 types, and confirmed the survival of each de novo subtype. Significant differences were observed over time [34]. These reported molecular classifications are mainly for serous cancer, particularly high-grade serous cancer of the ovary. Therefore, a more accurate molecular classification scheme for OC that can be applied to all histological types must be established.

In tumorigenesis, tumor microenvironment has essential regulatory function. The tumor microenvironment formed in the process of dynamic changes is regulated by various immunosuppressive signals, and its heterogeneity can lead to many aspects, including patient prognosis and treatment response [35, 36]. Recently, an increasing number of studies have found that tumor initiation and progression show close association with the microenvironmental factors surrounding tumor cells. In the expression of these genes, significant differences were found, indicating that the degree of immune cell infiltration in different metabolic subtypes is different and likely leading to differences in tumor progression and immunotherapy effects. At the same time, tumor-related cytokines and chemokines can recruit and polarize immune subpopulations and differentiate into protumor phenotypes, thereby promoting tumorigenesis.

In the present study, we tried to molecularly classify OC at the metabolic level and obtained new findings. Based on 2752 metabolic genes to classify OC, these samples can be divided into three metabolic subtypes (MC1, MC2, and MC3), which show significant differences in prognosis (Figure 2). Immune characteristics of different metabolic subtypes varied, which may also be related to differential responses to immunotherapy (Figure 4). In different research queues, metabolic subtypes are highly reproducible. An immune characteristic index is established based on metabolic subtypes, which can better indicate patients' immune characteristics as well as differential immune infiltration. A metabolic characteristic index is related to immune checkpoints. Moreover, we screened 11 gene markers potentially associated with the metabolic characteristic index, based on the coexpression network analysis. Among them, the differential expression of 6 genes, *GALNT5*, *FZD1*, *ZEB1*, *ZCCHC24*, *FBN1*, and *ITGA11*, showed significant significance for the prognosis of OC.

We have observed that NK cells and macrophages are highly expressed in various metabolic subtypes, while tumor-associated macrophages (TAMs) produce IL-10 and TGF-*β*. IL-4 and IL-13 are polarized to the M2 macrophage phenotype and support angiogenesis to drive tumor progression and recruit regulatory cells (Tregs) [37]. Poor cancer prognosis, including OC, is often related to accumulation of TAMs in the tumor area. Additionally, in the tumor microenvironment, CD8+ T cells produce IFN-*γ*, stimulating upregulated expression ofPD-1/PD-L1 and IDO1 genes [38, 39]. Studies have shown that upregulated PD-L1 expression in tumor cells, particularly when combined with PD-1 expressed by tumor infiltrating activated T cells, can induce exhaustion and inhibit the antitumor immune activity of these effector cells, thereby allowing tumor cell immunity to escape [40]. The upregulation of IDO1 expression is positively correlated with a poor prognosis and tumor progression and metastasis [41, 42].

In our study, we calculated the IFN*γ* score, immune T cell lytic activity, angiogenesis score, and immune checkpoint-related gene expression in the three metabolic subtypes. We found that the MC2 subtype with the best prognosis has higher T cell lytic activity and lower angiogenesis, IFN*γ*, and TIDE scores, indicating that this subtype has stronger immunogenicity and a good tumor microenvironment and is more likely to benefit from immunotherapy. In the differential analysis of immune checkpoint expression among the different subtypes, the expression of most immune checkpoint-related genes (LAG3, CTLA4, PDCD1, CD276, and HAVCR2) was significantly increased in MC1 patients with a poor prognosis. This finding implies that T cell exhaustion may exist in the MC1 subtype, likely explaining why MC1 shows higher immune microenvironment infiltration, but the prognosis is poor. Furthermore, the MC1 subtype is more sensitive to immune checkpoint inhibitors (CTLA-4 and PD-1 inhibitors) than the other two subtypes, further confirming this view.

Increasing evidence indicates that epigenetic changes play an important role in the pathogenesis of cancer. Many studies have reported epigenetic changes related to the clinical prognosis of OC, making the molecular classification of OC more complicated. Although the molecular prognostic evaluation model of OC has broad clinical application prospects and the relevant research results have been verified to a certain extent, no unified and widely recognized molecular prognostic evaluation model is available in clinical practice. Van de Laar et al. believe that OCs of early and advanced stages and different pathological types are different entities, and the biological behavior of the tumor, treatment option, prognostic factors and survival time are different, and different prognostic models must establish [43]. Presently, controversies exist concerning the scope of application of the molecular prognostic assessment model. All the established prognostic models are still in their infancy and require large sample verification and clinical application research. This is a huge task at this stage. When a prognostic model is not well applicable to new populations, the new data should be used to adjust the model first and recalibrated to improve its stability and adaptability. Only through verification-adjustment-reverification did the molecular prognostic model obtained in this way have reliable accuracy [44].

Studies on the molecular classification and individualized treatment of OC have only recently emerged. However, according to the current clinical evidence, molecular classification can be linked to individualized treatment and can become an effective method for OC treatment. In summary, the clinical diagnosis and treatment of OC in the near future will be based on molecular classification and prognostic evaluation based on molecular prognostic models, and then individualized molecular therapy will be performed, significantly improving the therapeutic effect of OC and improving the survival and prognosis of patients.

## 5. Conclusion

This study established a metabolic classification that can be used as an independent prognostic factor for OC and analyzed the differences in the characteristics of the tumor immune microenvironment among the different subtypes. Three subtypes performed significantly differential tumor microenvironment including immune cell infiltration and the expression of immune checkpoints, suggesting that the screened metabolic genes may play a role in immune modulation. In addition, three subtypes were differentially sensitive to immunotherapy, which could provide a guidance for assisting decision-makings in personalized therapy. Moreover, we identified 11 key genes that may closely correlate with the metabolic characteristic index, suggesting important roles of these genes in cancer metabolism. The 11 genes had the potential to be prognostic biomarkers for predicting OC prognosis, and these genes may also be the potential targets for understanding further mechanism of metabolic genes in OC development or therapeutic targets for treating OC.

## Data Availability

The dataset used and analyzed during the current study is available from the corresponding author on reasonable request.

## Supplementary Materials

Supplementary 1 Figure S1: (A and B) PCA chart before and after GSE data removal batch effect. (C and D) PCA chart before and after RNASeq data removal batch effect.

Supplementary 2 Figure S2: a heat map of the expression of 50 genes in RNASeq cohort.

Supplementary 3 Figure S3: CDF curve and consensus matrix in GSE cohort.

Supplementary 4 Figure S4: a heat map of the expression of 50 genes in GSE cohort.

Supplementary 5 Figure S5: Pearson correlation analysis between the expression of 11 genes and immune score (ESTIMATE) in OC and other 32 cancer types. The abbreviations of cancer types refer to TCGA and expression profiles of cancers were all obtained from TCGA.

Supplementary 6 Figure S6: Kaplan-Meier survival plots of 11 key genes.

Supplementary 7 Table S1: a list of 50 metabolic genes associated with prognosis.
