# Supplementary figures and images for: Construction of Metabolic Molecular Classification and Immune Characteristics for the Prognosis Prediction of Ovarian Cancer
Source: J Immunol Res. 2022 Jun 25;2022:2359349. doi: 10.1155/2022/2359349 (PMC9253871; doi:10.1155/2022/2359349)

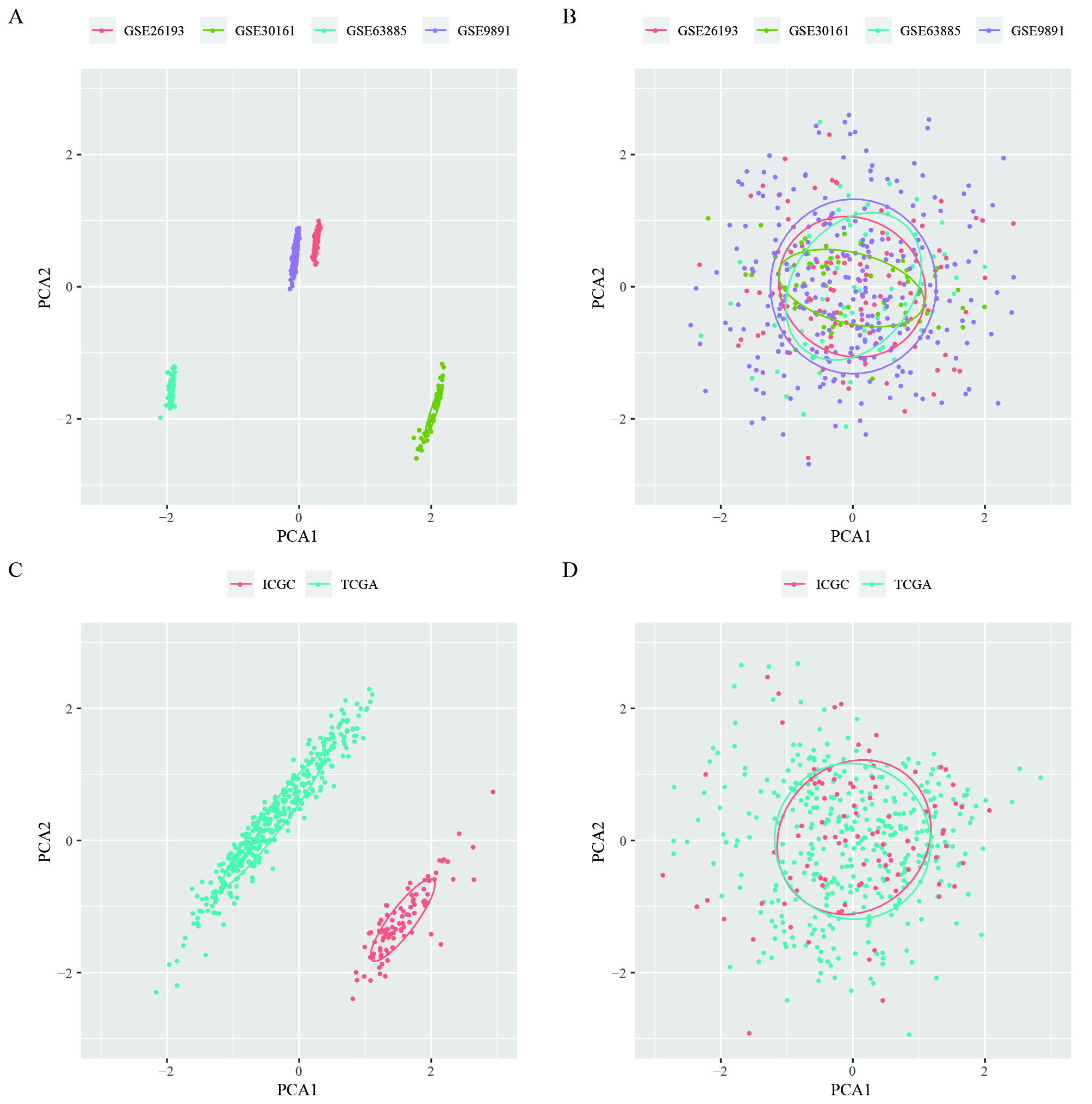

Supplement: Supplementary 1 — Figure S1: (A and B) PCA chart before and after GSE data removal batch effect. (C and D) PCA chart before and after RNASeq data removal batch effect. [file 2359349.f1.jpg]

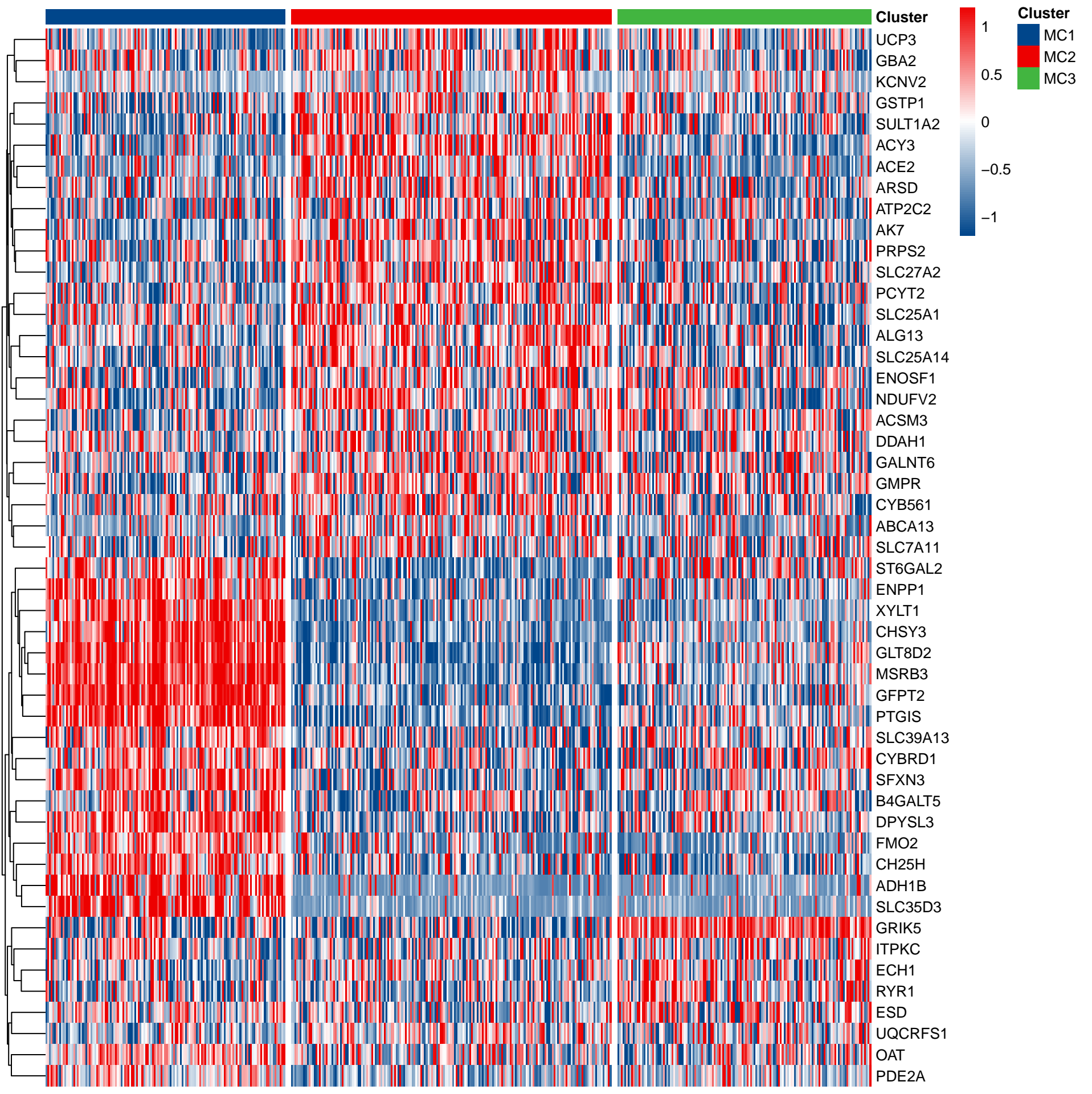

Supplement: Supplementary 2 — Figure S2: a heat map of the expression of 50 genes in RNASeq cohort. [file 2359349.f2.pdf]

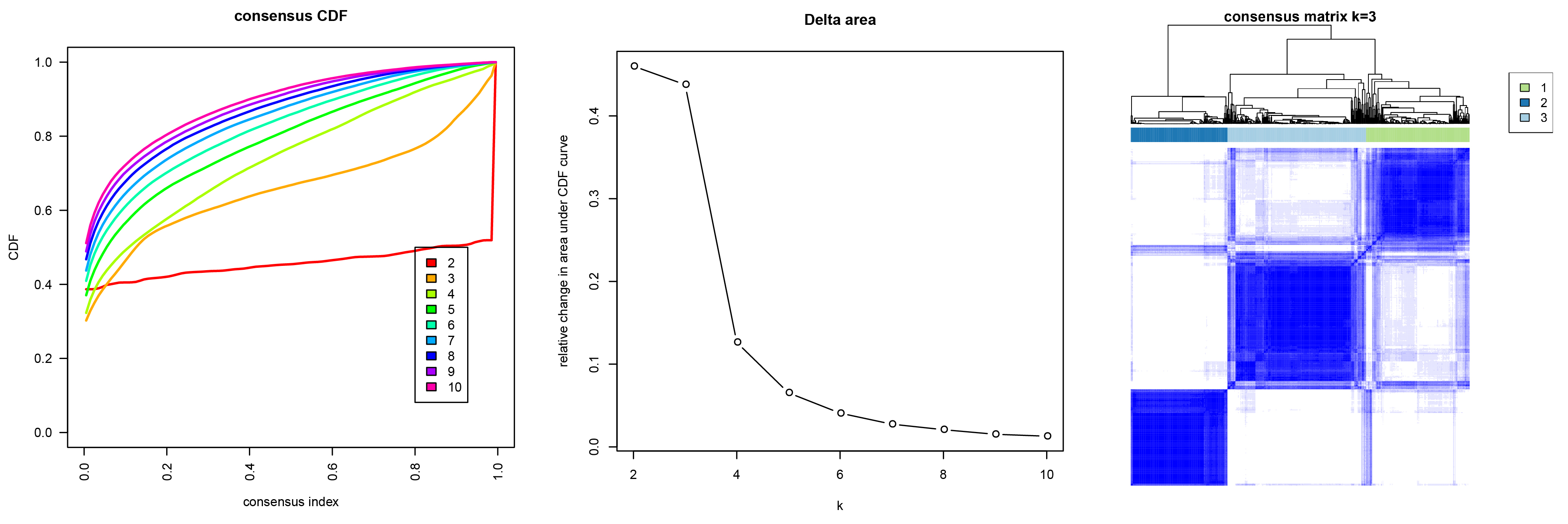

Supplement: Supplementary 3 — Figure S3: CDF curve and consensus matrix in GSE cohort. [file 2359349.f3.jpg]

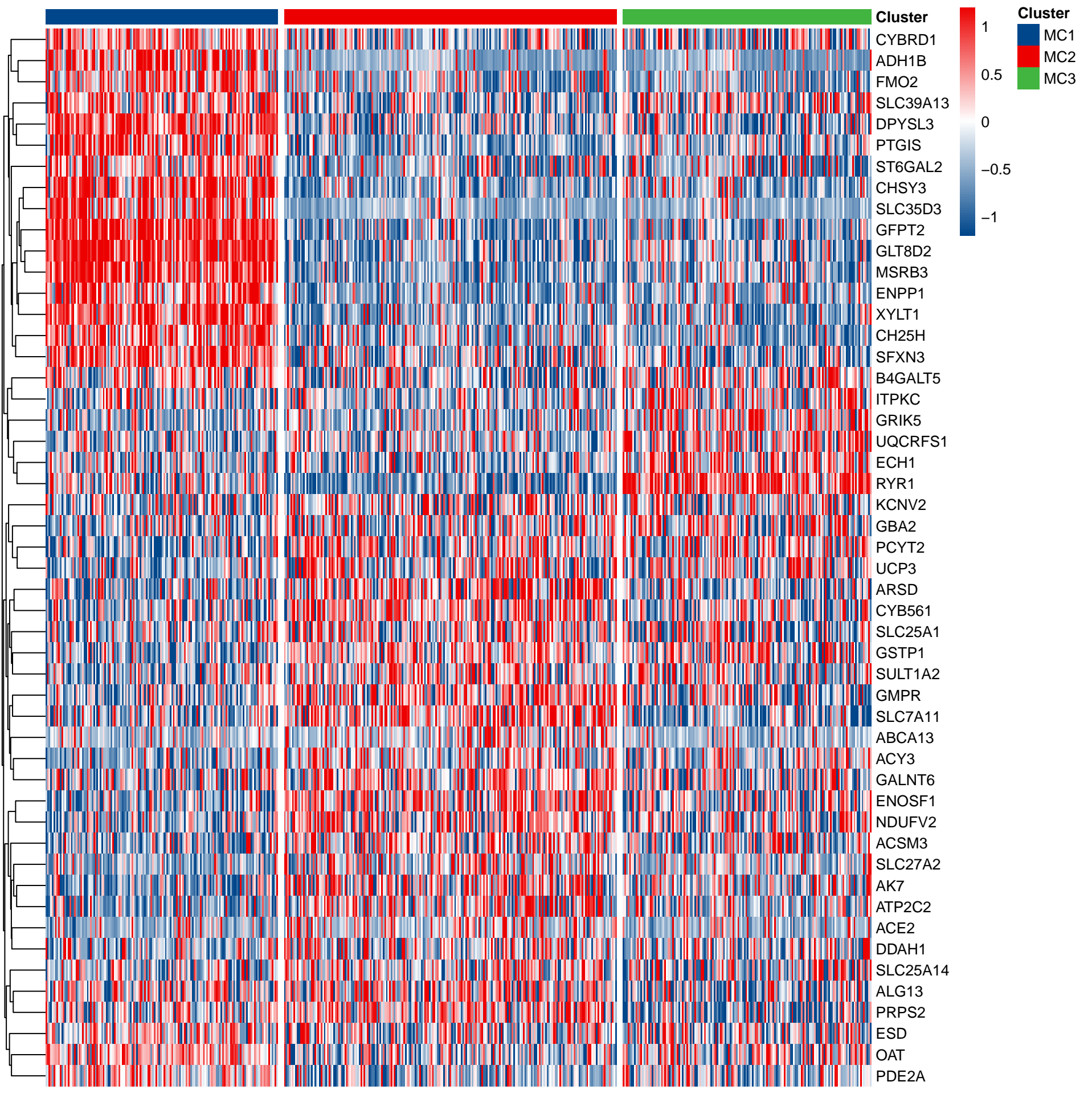

Supplement: Supplementary 4 — Figure S4: a heat map of the expression of 50 genes in GSE cohort. [file 2359349.f4.pdf]

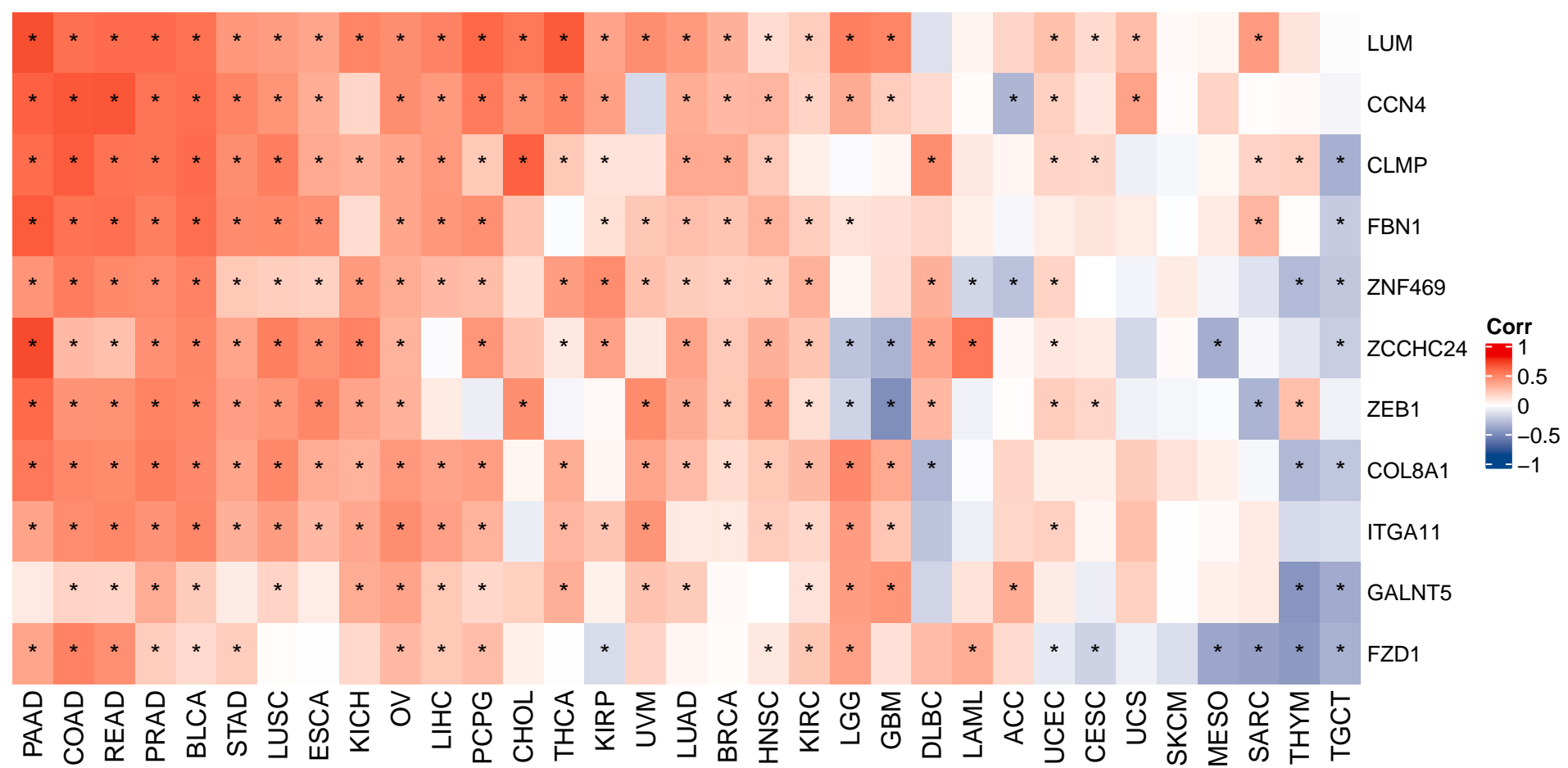

Supplement: Supplementary 5 — Figure S5: Pearson correlation analysis between the expression of 11 genes and immune score (ESTIMATE) in OC and other 32 cancer types. The abbreviations of cancer types refer to TCGA and expression profiles of cancers were all obtained from TCGA. [file 2359349.f5.pdf]

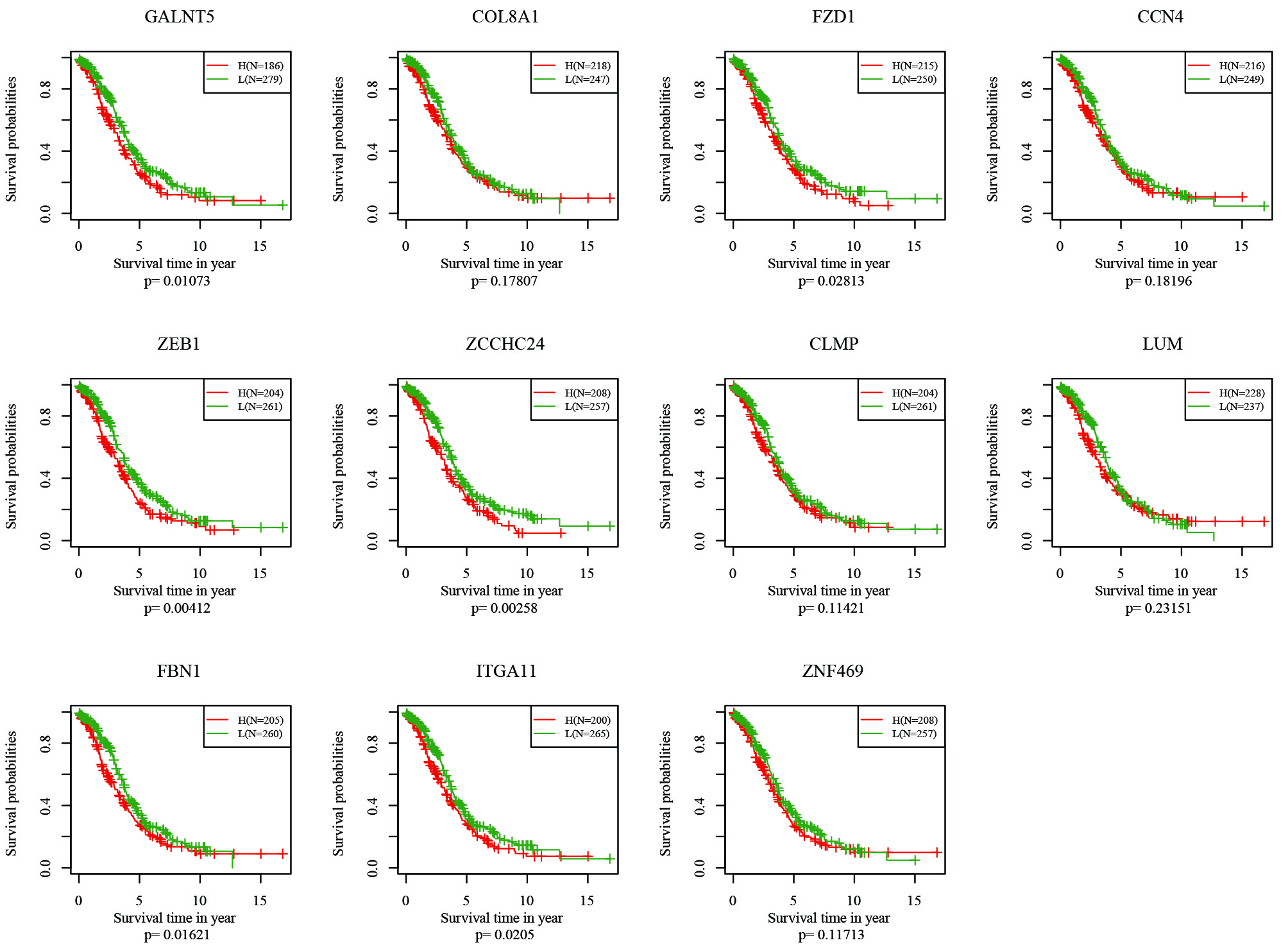

Supplement: Supplementary 6 — Figure S6: Kaplan-Meier survival plots of 11 key genes. [file 2359349.f6.jpg]
