# Supplementary material for: Construction of Metabolic Molecular Classification and Immune Characteristics for the Prognosis Prediction of Ovarian Cancer
Source: J Immunol Res. 2022 Jun 25;2022:2359349. doi: 10.1155/2022/2359349 (PMC9253871; doi:10.1155/2022/2359349)
Supplement: Supplementary 7 — Table S1: a list of 50 metabolic genes associated with prognosis. [file 2359349.f7.pdf]

Supplementary Table S1. A list of 50 metabolic genes associated with prognosis.

	genes
1	ABCA13
2	ACE2
3	ACSM3
4	ACY3
5	AK7
6	ALG13
7	ARSD
8	ATP2C2
9	CYB561
10	DDAH1
11	ENOSF1
12	GALNT6
13	GBA2
14	GMPR
15	GSTP1
16	KCNV2
17	NDUFV2
18	PCYT2
19	PRPS2
20	SLC25A1
21	SLC25A14
22	SLC27A2
23	SLC7A11
24	SULT1A2
25	UCP3
26	ADH1B
27	B4GALT5
28	CH25H
29	CHSY3
30	CYBRD1
31	DPYSL3
32	ECH1
33	ENPP1
34	ESD
35	FMO2
36	GFPT2
37	GLT8D2
38	GRIK5
39	ITPKC
40	MSRB3
41	OAT
42	PDE2A
43	PTGIS
44	RYR1
45	SFXN3
46	SLC35D3
47	SLC39A13
48	ST6GAL2
49	UQCRFS1

50 XYLT1
